# Wellbeing of School Communities in the Context of COVID-19 Pandemic: A Qualitative Study in Chilean Low-SES Schools

**DOI:** 10.3389/fpsyg.2022.853057

**Published:** 2022-04-11

**Authors:** Verónica López, Lorena Ramírez, Romina López-Concha, Paula Ascorra, Juan Pablo Álvarez, Claudia Carrasco-Aguilar, Pamela Jervis, Ana María Squicciarini, Ariela Simonsohn, Tabata Contreras, Héctor Opazo

**Affiliations:** ^1^School of Psychology, Faculty of Philosophy and Education, Pontificia Universidad Católica de Valparaíso, Valparaíso, Chile; ^2^Center for Research in Inclusive Education, Viña del Mar, Chile; ^3^Faculty of Health and Social Sciences, Universidad de Las Américas, Santiago, Chile; ^4^Department of Mediations and Subjectivities, Faculty of Social Science, Universidad de Playa Ancha, Valparaíso, Chile; ^5^Department of Industrial Engineering, Faculty of Physical Sciences and Mathematics, Universidad de Chile, Santiago, Chile; ^6^Millennium Institute for Research in Market Imperfections and Public Policy, Universidad de Chile, Santiago, Chile; ^7^Institute for Fiscal Studies, London, United Kingdom; ^8^Department of Programs, Junta Nacional de Auxílio Escolar y Becas, Santiago, Chile

**Keywords:** mental health, wellbeing, school, pandemic (COVID-19), Chile

## Abstract

The COVID-19 pandemic continues to impact schools and how education is conveyed to students. One of the aspects that has gained strength is supporting the wellbeing of educational communities. The purpose of this study was to describe and understand the construction of school wellbeing during the pandemic, based on the notion of collective and sustainable wellbeing. Through a qualitative design, we conducted a study in four Chilean low-SES schools in which a national school mental health program is implemented. A total of 41 in-depth interviews and one group interview were conducted with students, parents, teacher, teacher assistants, school principals, psychosocial professionals, and the school mental health officers during the second half of the 2020 school year. Thematic content analyses showed that, while facing the school closure challenges, schools strived to protect students’ and teachers’ wellbeing. However, participants highlighted necessary conditions for sustaining the school community’s wellbeing and mental health in the context of the COVID-19 pandemic: assuring digital connectivity for all students; coordinated work with families and within the school; strengthening networks; curriculum adaptation and diversified pedagogical strategies; and emotional support toward teachers, families, and students. We discuss these findings and their implications for a sustainable and collective perspective of the wellbeing of school communities in low-SES schools, as well as for policy, practice, and research from the perspective of schools for social justice and health promotion.

## Introduction

The COVID-19 pandemic continues to affect school communities and the way education is conveyed to students. According to the United Nations Educational, Scientific and Cultural Organization (UNESCO, 2020), the pandemic is the biggest school disruption in history. In this context, the World Health Organization (WHO, 2021) recommendations for schooling during COVID-19 highlights the relationship between the education and the health of children, and seeks for countries to recognize and strengthen this link by paying attention to the conditions and opportunities that are generated to promote social wellbeing and mental health of the different members of the school community (Duff et al., 2016; Velasco, 2021). In spite of these efforts, the COVID-19 pandemic threatens to perpetuate and increase inequalities and vulnerabilities, especially in lower income countries (Organisation for Economic Co-operation and Development [OECD], 2021).

At the beginning of the pandemic, most countries took multiple actions to close their schools and continue with the provision of educational services focused on curricular advancement and the use of technologies for distance and remote education (Brítez, 2020; Moreno, 2020). School closure has not been equal in depth and breadth between and within countries, with longer full or hybrid forms of school closure affecting students and families from lower-SES backgrounds (Garrido, 2020). The consequences of school closures include adverse effects on children and adolescent’s mental health and wellbeing, such as increases in an unhealthy lifestyle (Rajmil et al., 2021), symptoms of depression and anxiety (Meherali et al., 2021; Minozzi et al., 2021; Rajmil et al., 2021), and decreased life satisfaction (Rajmil et al., 2021). The impacts of the COVID-19 school closures on teachers have also been documented, and include burnout and stress (MacIntyre et al., 2020; Hascher et al., 2021; Pöysä et al., 2021). However, certain contextual aspects (e.g., school resources and collegiate support) as well as individual characteristics (e.g., coping strategies) have been identified as factors that help teachers to maintain their wellbeing (Hascher et al., 2021).

Teachers respond to students’ academic, physical, and emotional needs through positive teacher-student relationships (Jones and Kessler, 2020), which contributes to students’ wellbeing (Lavy and Naama-Ghanayim, 2020; Johnston et al., 2022). However, research on the ethics of care in the context of the pandemic has revealed that social expectations exist to a greater extent toward women teachers, who in a feminized career such as teaching, are often seen as those in charge of care and professional emotional management, playing multiple roles both for their own families and for the community educational (Rodriguez et al., 2022), which places the threat of increasing gender inequalities in teacher development (Kelleher, 2011; Gluz and Elías, 2020). In Latin America, digital connectivity and the economic resources that families own are worse and weaker in terms of availability, accessibility, acceptability, and adaptability (UN General Assembly, 1996) than those in Europe and Asia (Almazán, 2020; Reimers and Schleicher, 2020). This makes universal online or virtual learning practically impossible without adequate policies and resources (UNESCO, 2020), placing high levels of stress on teachers and leaving them to cope with their own individual resources for distance learning. Research in Latin American countries shows that teachers’ efforts, in many cases, have shifted from the teaching of disciplinary contents to the emotional containment and concern for the wellbeing of their students (Ramos-Huenteo et al., 2020; Sáez-Delgado et al., 2020). Perhaps all of these factors, jointly, might explain why Latin-American research has shown that distance teaching during the COVID-19 pandemic has been harder for female teachers, for young teachers, and for those who teach in low SES schools (Gluz and Elías, 2020; Salas et al., 2020; López et al., 2021a; Troitinho et al., 2021).

Although recent studies have contributed knowledge on the wellbeing of different actors in the school (Organisation for Economic Co-operation and Development [OECD], 2017), their development is still incipient, and the approach is mainly from quantitative perspectives of individual evaluation, or qualitative reports of student’s emotions and experiences (Scott et al., 2021). There are few studies that analyze the subjective experiences of the entire school community, but there is scarce evidence in the context of the pandemic crisis. In this study we understand wellbeing from a sustainability approach (Hargreaves, 2002; Shirley et al., 2020) and a collective perspective (Ramírez and Alfaro, 2018; Ascorra et al., 2021). The sustainable approach emerged in the 1980s (Suzuki, 2003) to refer to an interconnected system of durable human and natural value, where the survival and prosperity of species depends on maintaining the value of biodiverse environments (Hargreaves and Fink, 2000; Hargreaves, 2002). Applied to education, a sustainable approach asks about: (a) depth, referring to improvements that have human and moral value and support the learning and wellbeing of students and educators equally; (b) breadth, referring to improvements that extend beyond the individual level and highlight collective practices such as solidarity and cooperation; (c) length, referring to the prolonged duration of improvement over time with environmental impact; that is, improvements to students, teachers and schools that do not harm others; (d) diversity, refers to the non-standardization and homogenization of school processes; (e) restriction and renewal of energy, refers to the fact that changes should not overwhelm or exhaust human energies; and (f) conservation: refers to the recovery of the past to create the future.

The collective perspective of wellbeing aims to overcome the individual conception, which focuses on strengthening personal skills such as self-esteem and optimism to the detriment of collective skills such as cooperation (Ecclestone and Rawdin, 2016). Individual perspectives of wellbeing are criticized for overwhelming teaching and non-teaching professionals in schools to provide personalized attention to students, resulting in a predominantly individualistic culture (Gather-Thurler, 2004). In contrast, a collective perspective of wellbeing interprets wellbeing linked to the community, a territory, and a specific historical moment (Wyn et al., 2015; Ascorra et al., 2021). It also requires an organizational view of how to provide the conditions and opportunities for sustaining and strengthening the school community’s collective wellbeing through the interactions between the material/physical conditions, the deep values and moral principles, and the interactions between the different school actors.

Although scarce, there are a few studies which begun to study collective perspective of wellbeing during the COVID-19 crisis. Rivero and Bahena (2021) in Mexico, which questioned educational relationships and student wellbeing, emphasizing the interactions of the entire educational community. The study showed that general wellbeing is linked to the interaction of the different educational actors, and that this can be positive, even in virtual environments, in confinement, and in the midst of a crisis. In African countries, Ebersohn (2020) argues that a phenomenon he called “collective resilience” began to emerge. These are forms of spontaneous social and collective support within schools, aimed at promoting collective wellbeing. According to the author, the crisis contexts in Africa led people to mobilize different social resources, and in the scenario of the current pandemic, education is having a leading role from an intersectorial point of view. He argues that collective wellbeing in poor countries is only possible if support is activated in all directions and includes all actors in the school system.

### Context of This Study

The health crisis for COVID-19 surprised the Chilean educational system in the context of a social outburst known as the Chilean 2019 Spring, and in the transition toward a more inclusive State, through a new Constitution which is under draft, and a more inclusive education, with a recently passed Law on Inclusion and Law on Public Education, both with gradual implementation. Pre-pandemic, the implementation of both laws came into tension with a parallel context of high stakes testing through a nationally standardized testing system (SIMCE) with high consequences for low-performing schools, including school closure. Research shows that the translation of education as a human right and as social inclusion is made “the Chilean way” (Sisto, 2020), through a voucher system based on student attendance and high pressure on student’s achievement, that generates a consequent reduction of the curriculum focused on the high-stakes core subject areas of language and mathematics, and a tendency to apply differentiated treatments to low- versus high-performing students, with moral classifications of students as good or bad students based on their achievement (Ramírez et al., 2021).

The above context of the Chilean school system has been reported in the literature as a unique social space that allows analyzing market and accountability policies that pressure schools based on fear (Parcerisa and Falabella, 2017; Oyarzún, 2021). However, while it controls and monitors, the Chilean state also promotes educational policies that support schools in their wellbeing, such as the Life Skills Program (*Programa Habilidades para la Vida*, known as HpV). The HpV Program is a school mental health program that incorporates risk detection and prevention actions, providing pedagogical and psychosocial support that aims at vulnerable students, including support systems for improving school climate. The HpV Program is one of the world’s largest school mental health programs in breadth and scope (Murphy et al., 2017).

This ambivalence makes it interesting to investigate how wellbeing is addressed in such a critical context as the COVID-19. In Chile, one of the main public concerns during the current pandemic crisis in the education system is the “wellbeing of school communities.” This strong idea, wielded by both representatives of the College of Teachers and the Ministry of Education, comes into conflict when defining how to safeguard the wellbeing of school communities. During the COVID-19 crisis, the high-stakes SIMCE test and teacher evaluations have been suspended, but there has not been a policy of universal or prioritized access to technological devices and digital connectivity for lower SES students, leaving many students at the mercy of their families’ resources and time to reduce barriers to access distance education.

The situation improved during the last semester of 2021 when public schools were authorized to allocate the budget of their School Improvement Plans (Plan de Mejoramiento Escolar, PME) to buy cell phones, tablets, and mobile data plans for students. However, during the year 2020, which is when the interviews of this study were carried out, many students from lower SES schools were experiencing the digital gap (Centro de Investigación Avanzada en Educación [CIAE], 2020) and were not yet advancing to in-person teaching. As opposed to many European countries, where schools were the last to close and the first to reopen, in Latin American countries including Chile, hybrid forms of in-person classes did not begin to appear until 2021 with unequal progress in favor of private schools. This meant that, for most students in public schools, more than a year and a half passed before in-person schooling was again available for them.

The government’s strategy for the wellbeing of educational communities, and reducing learning gaps, also consisted of the creation of the “Aprendo en Línea” platform and a Digital School Library, without the consumption of the telephone data plan; the airing of the television signal “TV Educa Chile” to transmit educational entertainment content related to the curriculum of the first years of primary school; delivery of printed material in rural and vulnerable areas; a free Connectivity Solidarity Plan to provide internet for customers of some phone companies; the maintenance of food delivery by the JUNAEB (Public institution created by the Ministry of Education, responsible for providing support and scholarships to students) who supplies the School Meal Program; material for the socio-emotional accompaniment of teachers, students and families; and a proposal for curricular prioritization of learning objectives that was presented 3 months after the schools closed (Garrido, 2020; Belmar-Rojas et al., 2021).

In this context and based on the notion of school wellbeing as a collective and sustainable construct, the purpose of this study was to describe and understand how schools who cater for students from low-SES backgrounds generate and sustain the wellbeing of their school during a pandemic crisis.

## Materials and Methods

### Design

In this study, we used a phenomenological-hermeneutic qualitative design (Willig, 2008; Fuster, 2019) that allowed us to understand the meanings and practices in a specific and contextualized way from the perspective of the actors themselves (Denzin and Lincoln, 2003; Flick, 2015). Specifically, the four phases recommended by Fuster (2019) were carried out, allowing access to an in-depth understanding of the meanings surrounding the everyday experiences in the educational reality: clarification of assumptions of the research team, collection of the experience of educational communities in relation to collective wellbeing and the pandemic, reflections on the experiences lived with the people interviewed, and writing about this lived experience.

### Participants and Data Production Techniques

The study was carried out in four schools in the central area of the country (see Table 1). The criterion to choose each school was its theoretical relevance which was given by the context of the digital gap generated by the lack of connectivity of the students due to the lack of availability and accessibility of technological resources. These conditions were present mainly in lower SES schools. Therefore, we selected state-funded schools – public schools and privately subsidized – that met the following criteria: (a) the students come from families of low socioeconomic levels, (b) all the schools had the Life Skills Program (HpV), which is a psychosocial intervention program that incorporates risk detection and prevention actions providing pedagogical and psychological support for vulnerable students (Murphy et al., 2017).

**TABLE 1:** Participants and data production techniques.

Participants	Type of school administration	School vulnerability index (% of low-SES students)	Region	Participants interviewed	Individual interviews (N)	Group interviews (N)
School 1	Municipal	92%	Valparaíso	Principal	1	
				Teachers	3	
				Education assistant	1	
				Parents	3	
				Students	3	
School 2	Municipal	89%	Valparaíso	Principal	1	
				Teachers	3	
				Education assistant	1	
				Social worker	1	
				Parents	3	
				Students	2	
School 3	Subsidized private	94%	Valparaíso	Principal	1	
				Teachers	3	
				Education assistant	1	
				Parents	3	
				Students	3	
School 4	Subsidized private	94%	Valparaíso	Principal	1	
				Teachers	2	
				Education assistant	1	
				Parents	2	
				Students	2	
HpV program				Program officials		1
Total					41	1

We conducted a total of 41 individual in-depth interviews. The sampling was intentional, and sought to cover the diverse experiences, perspectives, ideas, and opinions of school principals, teachers, teaching assistants, psychosocial professionals, families and students, in the different school cycles of primary schools (which in Chile include preschool, elementary and middle school). All these actors were selected because they were characteristic cases of each school, since they had been working for more than 3 years or had a relationship with the school, participated actively in different activities to deal with the consequences of the pandemic, and agreed to participate in the interviews. Despite this narrow selection, this study sought to. To this end, we contacted the interviewees with the help of each school’s administration, and stopped collecting information when new themes ceased to emerge (see Table 1).

Table 2 shows the interview scripts for each type of school actor. As can be observed, interviews followed a general overall sequence in which we asked about participants’ general school experiences and emotions during the COVID-19 pandemic; classroom, school, and cultural/policy factors related to this experience; and their evaluation and future expectations.

**TABLE 2:** General interview scripts per actor with sample questions.

	Overall school experience during COVID-19	Classroom factors	School factors	Cultural factors	Evaluation and future expectations
School Principals	How has your experience as a school principal during the pandemic been?	During the pandemic, how has the learning process been conducted? What has worked well? What needs to be improved?	Regarding management, how has the school supported pedagogical and socioemotional needs? Which actor have played a key role in school management in this crisis context?	Regarding educational policies, what is your opinion -and experience- regarding how the health crisis has been managed? Concerning the management of this school, how has your experience been regarding the supports and pressures in this crisis context?	From the role you play in school, what have been the most favorable and unfavorable aspects you have experienced during this crisis context? > What do you expect should happen in the future?
Teachers	How has your experience as a school teacher during the pandemic been?	During the pandemic, how is the learning process being conducted? How was the planning, preparation of material and implementation of classes been conducted?	How has your relationship with the different actors of the school community?	Regarding educational policies, what is your opinion -and experience- of how the health crisis was managed?	From the role you play in school, what have been the most favorable and unfavorable aspects you have experienced during this crisis context? >What do you expect should happen in the future?
Teaching assistants	How has your experience as a teaching assistant during the pandemic been?	During the pandemic, how is the learning process being conducted? What is the role that teaching assistants had during the non-face-to-face period of classes?	How has your relationship been with the various actors in the educational community?	Regarding educational policies, what is your opinion -and experience- of how the health crisis was managed?	From the role you play in school, what have been the most favorable and unfavorable aspects you have experienced during this crisis context? >What do you expect should happen in the future?
Parents	How has your experience as a family been during the pandemic with this school and its activities?	During the pandemic, how has the learning process been conducted? What were the requirements made by the school, and how has this affected your family?	How has your relationship been with the different actors of the school community?	Regarding educational policies, what place does school take for you as a family in this pandemic? How was the experience of reconciling family and work with school life been?	From the role you play in school, what have been the most favorable and unfavorable aspects you have experienced during this crisis context? >What do you expect should happen in the future?
Students	How has your experience as a student been during the pandemic with this school and its activities?	During the pandemic, how has the learning process taken place? What works well? What needs to be improved?	How has your relationship been with the different actors of this school?	Regarding educational policies, what is your opinion about the general situation of the country and how this has affected the “school at home?” How has the experience of reconciling family with school life been?	From the role you play in school, what have been the most favorable and unfavorable aspects you have experienced during this crisis context? >What do you expect should happen in the future?
Skills for Life Program officers	How has your experience as an executor of the Life Skills Program during this pandemic been?	During the pandemic, how has the learning process been conducted? What worked well? What needs to be improved?	From the role you play, in what aspects have you been able to participate and influence the school management?	Regarding educational policies, what is your opinion -and experience- of how the health crisis has been managed? As for working at a school, how has your experience been regarding the supports and pressures to do your job in this crisis context?	From the role you play in school, what have been the most favorable and unfavorable aspects you have experienced in a crisis context? >What do you expect should happen in the future?

*Access to the complete version of the scripts is available upon request to the first author.*

### Procedure and Ethical Considerations

The interviews were carried out during August to October 2020 by one of the members of the research team through the Zoom virtual communication platforms. Interviewers agreed previously on the conditions and confidentiality protection. This agreement was supported by the signature of the researcher and participant of the letter of consent following the regulations of the Bioethics Committee of first author’s institution. All participants signed informed consent forms, and students signed informed assent forms. The most important ethical safeguards with the students were in the interview process, where we were careful not to ask questions that could revive fearful experiences that could induce re-traumatization (Watson et al., 2022). We asked them to tell us about their needs through simple examples that would allow us to describe a common day, and we avoided delving into experiences of great discomfort. In addition, we emphasized that if they wanted to withdraw or call their mother or father, they were completely free to do so whenever they wanted. The results of this study produced inputs for the HpV Program to promote the wellbeing of school communities in the COVID-19 context as part of its programmatic actions, and therefore, the main results of this study were informed to the program’s central and regional teams.

### Data Analysis

Data were analyzed using content analysis. This analysis responded to the phases of content analysis of the phenomenological-hermeneutic method. First, successive readings were made of the entire corpus and then, preliminary coding was done for each actor in order to have a manageable subset of data (Fuster, 2019). To do this, we organized the information around the themes of the interviews, assigning emergent codes within each theme. We constructed a total of 213 codes. Those that had more associated textual quotations were categorized by grouping them into large common themes. We did this procedure within each group of actors (teachers, families, students, etc.) and then, we contrasted them in order to have a global and broad look at the themes in a cross-sectional way (Denzin and Lincoln, 2003; Flick, 2015). In this way, we obtained six broad and common categories, some of which are more represented within some actors (see Table 3).

**TABLE 3:** Analytical categories per type of school participants.

Categories	Participants					
	Principals	Teachers	Psychosocial professionals	Parents	Students	HpV
Assuring digital connectivity for all students	x	x				
Coordinated work with families	x	x	x	x	x	
Coordinated work at school	x	x	x	x	x	
Strengthening networks	x	x		x	x	
Curriculum adaptation and diversified of pedagogical strategies	x	x		x	x	
Emotional support toward teachers	x	x	x	x	x	x

## Results

Results from the thematic content analyses showed that, when in the face of a major crisis, schools strived to protect students’ and teachers’ wellbeing. During the course of the interviews, however, participants revealed that certain conditions are needed to sustain the safeguard and wellbeing of the school community in the present and future. Table 3 presents the general categories per type of school participant. The results show six dimensions that characterize, from the voices of the school community, the necessary conditions to promote and protect their wellbeing in the context of the COVID-19 pandemic: (i) assuring digital connectivity for all students; (ii) coordinated work with families; (iii) coordinated work at school; (iv) strengthening networks; (v) curricular adaptation and diversified pedagogical strategies; and (vi) emotional support toward teachers, families, and students.

### Assuring Digital Connectivity for All Students

One of the most important aspects raised by principals and teachers in all schools was the importance of promoting the conditions to ensure digital connectivity of all students. In this regard, one of the principals pointed out that one of the main problems was that in her school, the students come from families with low economic resources, so they do not have the means to pay for internet connection:

*“We have a vulnerability index, the last one given to the school is more than 90%, therefore, it is very high, the parents do not have connectivity, and generally, there is only one cell phone, and the mother who works or the father who arrives in the afternoon uses that cell phone, and that is the reality.”*

(School Principal, School 3).

While schools remained closed, the internet system became a fundamental tool to continue remote and distance education. In this sense, the emphasis was placed on the importance that all students, as well as the different actors of the school community, had access and good connectivity to promote the learning and “not some more than others.”

*“I believe that* [sighs] *obviously in this way, strengthening all the shortcomings that we are encountering along the way, having better connectivity, that we all have access, not that some more than others, eh, that they take us all the same, in the sense that, without seeing who is a teacher, who is a principal, who is an assistant, but that we are all a contribution and that we have the same, eh accesses, eh. and obviously strengthening everything in which we are a little weaker, to be able to support the parents more* (.) *so that they too can, eh, have, eh, the possibility of being able to participate in this and that their children learn.”*

(School Principal, School 2).

To ensure connectivity, schools reorganized the available resources with the cost of running out of budget for other activities:

*“Today we are working on our school improvement plan with the purchase of tablets for all students, the 434 students of our school, plus a Bam, which is a modem for connectivity for the 434 students, that meant running out of money, without resources, for other initiatives that were proposed or that I was thinking of a-, initially, but as long as I cannot connect with my students I cannot do any other activity, everything else is postponed and here there is a basic need that must be met.”*

(School Director 2).

Based on this new reality and conditions, school principals and teachers mentioned that progress must be made toward improving connectivity for remote and distance education, the management of ICTs, and technological implementation, allowing adaptation to the new future virtual scenario. In this regard, a teacher points out:

*“So maybe I don’t know if as a government I would say hey, just as they have guaranteed food, they have guaranteed connectivity, in the same way that students here in Chile have a reduced transport fare, they could have reduced connectivity* [fare] *up to high school, and even college students, they could have reduced connectivity* [fee], *to me that is a question that kept me spinning, that is, the only way that we can have and make sure that the child has access to education is that he is only connected, it*’*s the only way.”*

(Teacher, School 2).

### Close Coordination With Families

Another aspect mentioned by the different actors of the school was the importance of maintaining a close relationship and coordinated work between the school and the families. The coordinated work became essential in the context of a pandemic since contact with students, especially those who are in pre-school and primary, is through parents and/or guardians. This contact implies a new challenge for the schools, since it means, first, knowing the reality that each family is experiencing:

*“We began to interview each of the parents, we called each of the parents by phone, we made WhatsApp groups of the classes with all the parents, once we implemented this survey and more or less, we knew how many people had access to the Internet and who did not, we began to work differently, we began to record videos.”*

(Special education teacher, School 2).

This first task not only implies having a comprehensive view of the reality of the families but also of each of the members of the school staff. In all schools and to all types of school participants (see Table 2), this contributed to the collective sense that *the school cared* and that families were not going to be left alone:

*“Before starting with the printed material, the first thing that was done was a work of connection with the families via cell phone. We spend all the minutes, all the* [digital] *plans of all the school staff* [which were self-funded by each staff member] *eh, the* [psychosocial] *duo* [school psychologist and social worker] *and the [director’s] team, calling the families and calling the staff themselves, the teachers themselves, the workers themselves, that is, we considered that it was important that to move forward, we had to have our workers connected and feeling that we were worried about them, it was the first thing, and secondly, for the families, that they saw that the school had not left them alone.”*

(School Principal, School 2).

Therefore, for the schools, one lesson learned from the health crisis was that a necessary condition to favor the learning and wellbeing of the students was maintaining close and permanent contact with the families:

*“Well, I think it has to start from generating spaces of trust, starting with that. Because, for example, I have always tried to the students and the parents “whatever you need, you just call us,” and this was achieved at the beginning* (.) *This was a whole process, at the beginning, a process of adaptation. Now I believe that it is necessary to start from the base of generating trust, of understanding that even though we do not know each other that much because we are all new at the school.”*

(Teacher, School 1).

Families highly valued the fact that schools maintain permanent contact with them and attend to their different needs. For them, “closed contact” means establishing an open-school system that allows them to make consultations and receiving guidance from teachers to provide better support in their children’s homework:

*“Once a week the school is open for all kinds of inquiries, or especially now to the, for everything for the eighth year that I had to apply for, for the high school because, sometimes, and even the school is the one that looks for the parents who forget to apply for the children* [referring to the School Admission System for entering high schools in schools who are only k-8].”

(Parent, School 4).

*“More has happened to me in mathematics, that has been hard for me, they say they are ready eh exercises and they are badly done, but eh I had to correct this and that and if you* [as a parent] *don’t know how to do it, she (the teacher) writes me, in fact she even sent me a draft in a notebook so that I could understand it better, and the fact is that I understood it better and in this way one* [as parent] *can explain it* [to his/her child].”

(Parent, School 4).

In the same way, students recognized the efforts and concern of their schools in using different strategies to ensure that students could access and remain in school:

*“The school has also been very concerned about students who do not have internet to attend classes. They have a transport that goes to the community, for example, from here in Cartagena where I live, to Quisco and El Tabo, which will deliver guides so that the students are not totally lost.”*

(Student, School 1).

### Coordinated Teamwork at School

Another of the conditions necessary to promote the wellbeing of the school community was coordinated teamwork at school. This aspect was especially raised by school principals, school staff, parents, and students, who argued that holding periodic meetings of the management teams with other school-level stakeholders as well as knowing the opinions of the entire school community are a key aspect to making consensual decisions that are pertinent to the changing needs of the health crisis:

*“In fact, we had the last meeting with the school council, which we are also complying with that rule of the four* [annual] *meetings and everything and sending it to the provincial* [school district authority]. *And we had the president of the student body give us super good ideas, she said “I can also cooperate,” and that is something that we did not exploit, the capacity of the students.”*

(School Principal, School 3).

In this regard, students positively value when teachers generate the instance to talk with them about education in the current times of pandemic:

*“There are some teachers who there is nothing to say* [meaning they are great], *because yesterday, at least with the principal and some of the technical staff that there are in the school, they called me and some other classmates for a meeting similar to this one, to talk about the, the education in the pandemic*, [to talk about] *teachers, to talk about ourselves, about the vision that we have as students, about the online classes. On what things we could contribute to make the classes better.”*

(Student, School 4).

Simultaneously, the importance of collaborative work between teachers is emphasized, which allows maintaining good working relationships, as well as benefiting from the support of their peers, especially in the new remote and distance education scenario with the consequent use of ICTs:

*“Other colleagues, it has been, uh, super good because we had like the time and, of course, now that we are on the subject of social networks, one asks the other “hey, what did you do? How did it work? Look, you know I have this idea, I want to do something”, and between conversations, we support one another. Among colleagues, at least, the support is working super well, eh, the coordination we have between us is working super well.”*

(Teacher, School 2).

Another aspect that is central to schools is the fact of strengthening coordination with the psychosocial teams of the schools. In Chile, the psychosocial team is the name given to the coordinated work of school psychologists and social workers (López et al., 2021a). Coordinating with them is known as especially relevant to meet the various needs and demands of the pandemic:

*“The difficulties that each of the families present, we now have, great support is formed, eh, psychological, of school climate* [team], *we have a counselor. This year created something that, was not as strong before and now it is happening, and we are caring a lot for all the families who are with many problems, because there are many cases that we do not even know what is happening, so it is about reaching out the largest number of people.”*

(School Principal, School 1).

Parents helping each other is also recognized as a facilitator:

*“For example, many parents don’t have* [access] *to connect to YouTube, but it is not only for example to share the videos among us, that is, we download it, we send it to them, and so they can have access.”*

(School Principal, School 4).

Finally, students also recognize how important it is to maintain the bond and contact with their classmates as a way of helping and accompanying each other:

*“Among friends, eh, we talk so as not to lose communication, which is the most important thing. Because, with the issue of the pandemic, the communication, I, in many cases it has lost a lot, a lot of strength. Because we are all locked up, and the. And the physical contact that may exist, which existed before when classes did not exist, right, it’s not there now. So, one must cope by talking to people, not losing contact and that is what we do.”*

(Student, School 4).

### Strengthening Social and Emotional Support Networks

One aspect highlighted by schools to favor the wellbeing of families and students in the context of a pandemic is related to generating and/or strengthening social and support networks with other ministerial and local programs. The articulation with networks is mentioned, not only through work done by psychosocial pairs who can focus on vulnerable situations but also through the support that the Ministry of Education and the municipalities can provide to schools:

*“In other words, here the support networks for the schools are fundamental, the work that* [names the HpV Coordinator in the school] *does with his people, the work that the Ministry will have to do as well, the work that the municipalities have to do, that is, we will have to unite all these positive forces to make this happen.”*

(School Director 1).

In this sense, one of the contributions most mentioned by the management team was the support received by the school mental health program (HpV) and by the drug prevention program SENDA, as well as the work carried out by the school psychosocial teams:

*“HpV on one side, SENDA is also helping us in the same way, which is in* [emotional] *containment, the DAEM* [municipal authority] *has a unit that deals with psychosocial issues that also supports us* [although] *more, I would say farther away than close, but they do things, and the other for example, is the work that my psychosocial duo is doing, right? So, we maintained this contact with SENDA, HpV and we are quite good, the material that arrived is good, eh. I hope, I hope that my colleagues can also implement it.”*

(School Principal, School 1).

This support from the networks is also perceived as very relevant for teachers, since they recognize that the activities generated, especially by the HpV Program, allows student to access other types of social and emotional supports:

*“Look, I think there is something super important, very important, at least that I, I insist, I value it very much, and it is the HpV program.* (…) *When they come to school it’s like, to the courses, it’s like “oh, how delicious!” and “how beautiful the activity was” and suddenly they leave them* [emotionally, cognitively] *touched.”*

(Teacher, School 3).

Likewise, from the perspective of the parents and students, one of the aspects most mentioned was the management and coordination that both the schools and the municipalities carried out to ensure that families receive the States’ public-school food for lower SES students:

*“We have received here at home, we have been receiving the* [food] *box from the JUNAEB* (National Board of Student Aid and Scholarships), *and, the municipal truck also delivered, passed by delivering merchandise.”*

(Parent, School 1).

*“We received it, yes, yes, and they also made like another box of merchandise when eeh they knew that one did not, because they were pending if they had eeh, what’s it called, parents working, some were left without work and they supported them in giving them another box of merchandise, they came to leave it at your house, pending if something was missing, and all.”*

(Parent, School 1).

### Curriculum Adaptation and Diversified Pedagogical Strategies

Curricular aspects have been a central aspect that schools must rethink to generate the best conditions for remote and distance learning for all students. In this sense, schools reflected on the amount of content required by the national curriculum and prioritized those contents that they considered essential:

*“So, we are talking about the fact that today the Chilean curriculum is extensive and could definitely, eh, certain essential learnings are prioritized over others that are not so essential, because they are contained in the previous ones, Ehm, they are more inclusive, umm, they are more integrative. So, under this view, indeed the school to come will be completely different from the school that is and the one that existed before.”*

(School Principal, School 2).

In this context, one of the crucial tasks for the management team and the teachers is to generate the curricular adjustments that allow delivering the necessary and pertinent contents to this new educational reality:

*“But all the students have a job that is equivalent to four weeks with two guides per subject, uh, prioritizing language subjects, we prioritized, in the first instance, as a school, without* [previously] *receiving ministerial instructions, that we were going to prioritize language, mathematics, science and history, and in the last two years of high school, the modules that are essential for technical-vocational track, which are approximately two to three modules for each specialty, no more, not the 6 or 7 that* [usually] *exist.”*

(School Principal, School 2).

In turn, school staff considered necessary to rethink the most pertinent pedagogical strategies to the situation, especially considering that for many students these new educational conditions affected their motivation. In this sense, apart from being able to ensure the technological conditions that allow remote and distance education, school staff emphasized the importance of generating educational scenarios in which the students can make inquiries and talk about their experiences. However, they recognized that this was not always achieved:

*“Somehow, we are not comfortable with what online classes are because, there is very little space for the student to talk with the teacher. Not exactly about a subject, because, although that is what classes are supposed to be for, you still need the space to talk about any type of subject and, and for the conversation to come out of how one exactly is doing* [meaning], *how the student feels.”*

(Student, School 4).

This is an aspect that was also mentioned by parents, for whom it is necessary to incorporate more recreational activities, which in some way compensate for the confinement and lack of activity of the students at home:

*“Therefore, due to so much confinement, an activity like interactive, which can make them, like, play, I don’t know, as if they were in classes, as if they had an equal* [peer], *they are children, therefore an interaction, like in games.”*

(Parent, School 1).

From the parents’ perspective, teachers must maintain a close relationship and a diversity of responses to the educational needs of their pupils to facilitate their learning process:

*“For example, if he* [her child] *has problems in math, she* [the special ed teacher] *tells him, ok let’s see, show me the exercises they sent you, he sends the exercises to her and she explains how by video call, or now they also use Meet which is the same as Classroom, she explains to him how he must do it. But she is always aware of him, she is always calling him”*

(Parent, School 1).

### Emotional Supports for Teachers, Families, and Students

One of the aspects most highlighted by all the interviewed participants was the need to generate different spaces of containment and emotional supports directed toward teachers, families, and students. This is because the health crisis has different effects on people’s lives and schoolwork:

*“The* [psychosocial] *pair, from the point of view of containment, was initially working by telephone, calling person by person, student by student, parent by parent, each one of the school staff, right? What was the objective of this first connection? containment. It was that, bind and contain, nothing more. After that, when we started with this pedagogical material work plan, we also began to advance with the [psychosocial] duo, and what do we start doing with the duo? Begin to focus certain cases over others*, (.) *we began to focus according to certain needs, and then there began to be a monitoring of certain families in specific for violation of rights.”*

(School Principal, School 2).

A significant aspect in this sense was to provide containment and support toward teachers, given that one of the situations that they usually experience is demotivation in the context of remote and distance teaching with turned-off screens or non-connected students.

*“So I always tell them “but calm down, they must have this ability to understand that it is not easy for anyone, that is, it is not that the student devalues your work, but simply maybe he fell asleep,” because, of course, in this remote context there is no student in the room, where control is held by one, the teacher, and that is the teacher’s greatest deficiency, in the sense of understanding, not everyone has the ability nor capacity for change, not everyone has the capacity to be flexible.”*

(School Principal, School 2).

*“From the psychologist and the social worker, who are the psychosocial team, we had, that is, received eh, like these tips, right, so that we don’t get overwhelmed although we know that the same is. Let’s see, it’s something that has to do with a process, it’s not like I say overnight “I’m not going to get overwhelmed” and it goes away, isn’t it, right? But, I mean, there are instances*

[designed for them as teachers].” (School teacher 4).

For parents, it was also essential that some spaces and activities exist to allow students to distract themselves and forget about the daily worries that families experience:

*“But I still believe that there are children who may, like they need, for example, something to distract them, something to get them out, as I told you something to get them out of their world because they still, they are so like locked up that they need something, that makes them forget their worries or what, they are so involved in their parents’ concerns.”*

(Parent, School 1).

Similarly, for students, it was very relevant that teachers maintain closeness and were interested in their lives and situations and not only dedicated time to imparting content during class:

*“That’s where I tell you that it depends on the teacher, because I can take my hat off for a teacher, that she is one hundred percent worried about us, she takes the time to be able to talk with us, to ask us how is the, our situation before starting the class and directly starting to pass the subject. She cares about us. But there are teachers also in another case, at the other pole, who only start class, start recording and begin to explain, explain, explain and don’t give the time to perhaps think that the student is not understanding them and pause and be able to explain exactly what the student is not understanding.”* (Student, School 4).

Finally, the support and containment toward the students was a primary task for the implementers of the HpV Program, from which they adapted their intervention strategies to better reach the students: for the team, one of the main tasks to perform in the context of a pandemic is to generate activities with students.

*“We have had difficulty to generate these virtual encounters, and therefore, we made the decision to continue with this, to intervene in the same way with the children, more than anything in terms of containment, to support them, in what their own process is also with the pandemic, and also consider its dimensions of risk.”*

(HpV Group Interview).

## Discussion

The COVID-19 pandemic continues to impact low and lower-middle-income countries and the most disadvantaged children and adolescents in each country. While protecting a population from the ravages of the disease is clearly important and even more so when the health infrastructure is poor, a key question is how to address the impact of the pandemic in school communities and how education is conveyed to students, which if left neglected can have dramatic long-term consequences for the wellbeing of school communities.

The Health-Promoting School (HPS) approach proposed by WHO more than 25 years ago seeks that countries and schools recognize the relationship between education and health and promote principles of equity, sustainability, inclusion, empowerment and democracy (Velasco, 2021). This perspective considers that all aspects of the school have an impact on the health of students, and at the same time, that school is so much more than academic learning. Therefore, the school should educate in the promotion of physical and mental health, as well as social wellbeing. The school has an obligation to provide a healthy environment through the implementation of structured and systematic policies and action plans that consider the holistic experience of students, teachers, and non-teaching staff. The HPS model a flexible model that adapts to the characteristics and culture of schools and countries. The model proposes that a health promoting school environment is one in which there are policies for the promotion of collective and integral health of all school members; a physical environment that includes buildings, grounds and school surroundings; a social environment that caters for the quality of the relationships between school community members; individual health skills and action competencies, like healthy eating, daily physical activity, developing social skills and health literacy; community links; and health services collaborations (Velasco, 2021).

**FIGURE 1:**
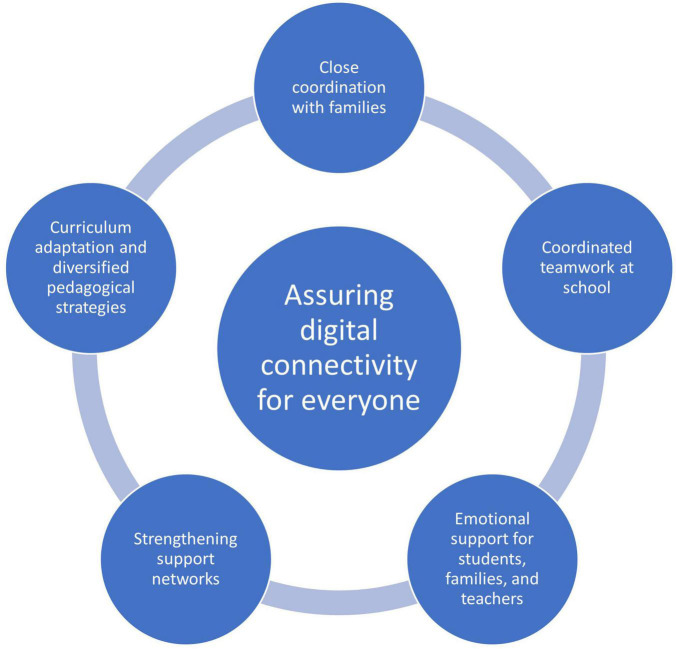
Conditions for a collective, sustainable wellbeing of school communities during the school-closure period of the COVID-19 pandemic: Summary of research findings.

Findings show that school principals and teachers conceive the digital gap as a barrier to students’ and their families’ wellbeing. Therefore, digital connectivity during the COVID-19 school closure period of the pandemic is viewed as a basic condition for school wellbeing (see Figure 1). Following the HPS model, the digital gap was conceived both as lack of adequate physical environment, as well as of equitable educational policy. In their discourse, participants raised issues of availability, accessibility, acceptability, and adaptability of technological resources necessary for distance education were raised. These “4 A’s” form part of the United Nation’s (1998) criterion for assessing the State Parties’ implementation of the right to education as a human right. Therefore, in the context of the COVID-19 pandemic, the findings of this study suggest that school staff consider digital connectivity as a crucial part of the right to education. However, their discourse also considers a quest for inclusion: assuring digital connectivity for all students and also for school staff (Ainscow, 2020). In this regard Chile’s educational policy during the pandemic did not plan nor implemented a universal digital access policy to the decentralized school processes, nor, during the first 6 months, provided extra budget. Rather, publicly funded schools had to use available resources and budgets meant for other purposes. Participants felt a lack of support from the Education Ministry and municipalities, but at the same time they highly expected this support. The educational communities considered that public policy did not provide responses with the urgency required, nor dealt with educational inequality. This perception of lack of support may imply a deep drain of energy that can lead teachers, students and their families to experience mental health difficulties. Although existing public psychosocial support programs which follow the WHO approach, HpV and SENDA, are positively valued in the context of the pandemic, it seems that schools expected more from their authorities, especially from government authorities.

Three other conditions for school wellbeing that were raised by participants are theoretically aligned with HPS’s recommendations for providing a quality social environment, community links, health services collaborations, and developing individual competencies. Findings show that, in the face of the current health crisis, the primary concern of the educational communities was mutual support and the wellbeing of everyone (Ebersohn, 2020; Rivero and Bahena, 2021). The strategies for achieving this was through close connection and coordination with families; coordinated teamwork at school; emotional supports for students, teachers, and families; and by strengthening support networks (see Figure 1). The close connection and coordination with families, which had not been as present pre-pandemic, was a major objective during the first half of the first year of the pandemic with required major efforts in the case of the digitally non-connected students. With time, sense of accomplishment in connecting and working with families helped widen the sense of the “school community”. In the schools studied, this was done first by drawing the school staff closer together through what they called coordinated teamwork, and then working coordinately to connect with families in order to reach out and engage students. The literature on school connectedness suggests that these kinds of strategies facilitate positive student-teacher relationships (Zullig et al., 2011) which are highly relevant for school belongingness (Allen et al., 2018) and students’ wellbeing (Chu et al., 2010), while at the same time protecting students from engaging in risky behaviors (Drolet et al., 2013), and preventing school dropout (Zullig et al., 2011). Probably, these kinds of local strategies might partly explain why the school drop-out rate did not increase after the pandemic school year of 2020, considering the previous year rate (Rivero and Bahena, 2021).

We conclude that the school communities’ discourse is aligned with a perspective of sustainable and collective wellbeing. On the one side, and in line with the theoretical assumptions of this study, the schools participating in this study emphasized the collective character of school wellbeing during the pandemic. Phrases such as “the student is not alone in the classroom”; “it’s not just about the students’ wellbeing, it’s also about our teachers’, school staff’s and families’ wellbeing” were repeated throughout the interviews. Certainly, the lack of digital connectivity throughout the school closure period placed a great amount of stress on teachers and school staff (UNESCO, 2020). In order to cope, the contextual aspects such as school resources and collegiate support identified by Hascher et al. (2021) have helped teachers maintain their wellbeing (Hascher et al., 2021) and respond to students’ academic, physical, and emotional needs through positive teacher-student relationships (Jones and Kessler, 2020). The findings of the present study are consistent with López et al. (2021b) findings that students’ wellbeing is permeable to their perception of their teachers’ wellbeing (Shirley et al., 2020). On the other hand, the results show that students in the context of a pandemic have valued even more the presence and closeness of their teachers, highlighting as relevant being able to feel cared for and welcomed in their needs. Consistent with the literature reviewed, these results show the high demand that has fallen on teachers, which, as we pointed out, is strongly feminized (Kelleher, 2011; Gluz and Elías, 2020), who have had to take on care roles toward their families as well as toward their students, with a strong impact on their own wellbeing and health (Salas et al., 2020; Troitinho et al., 2021; Rodriguez et al., 2022).

The ways in which the participants of this study construed a collective notion of school wellbeing, and the different forms of connections, coordination, and collaborations through which this took place, is similar to spontaneous social supports that Ebersohn (2020) suggests have emerged in African educational communities at the beginning of this pandemic (Ebersohn, 2020), and to the closely knitted interactions of the entire educational community that Rivero and Bahena (2021) observed in Mexico. Perhaps, as these authors pose, in the context of weak national or regional-level policy supports which are frequent in developing countries, the COVID-19 crisis has led schools to mobilize different social resources from different actors and in many directions, granting education a leading role from an intersectorial point of view (Ebersohn, 2020). As Vélez (2011) points out, in the context of crises, societies expect schools to restore hope and stability. In line with Hascher et al. (2021) findings, this study provides qualitative evidence that this is being done through carefully planned day-to-day coordination among the school staff, with families and with existing support networks found.

Recent research on school during the COVID-19 crisis in Latin American countries shows a shift from teaching disciplinary contents to the emotional containment of their students (Ramos-Huenteo et al., 2020; Sáez-Delgado et al., 2020). In effect, many participants in this study highlight emotional containment as a major strategy given student’s and families’ emotional needs during the pandemic, as a consequence of vital changes in family economy and due to the health-related issues of the crisis. What this study shows is that teachers’ and school staffs’ spontaneous responses to students’ emotional needs through reaching out and listening to them, when coordinated and supported by available support from in-school and out-of-school mental health services, can promote the school community’s collective resilience (Ebersohn, 2020) and help prevent serious mental health issues. However, this study also shows that the educational side of the HPS approach during crises must also be dealt with. In line with other qualitative studies of students’ experiences during COVID-19 in other parts of the world, our findings suggest that one of the biggest struggles for children and youth has been remote learning and maintaining academic routines (Branquinho et al., 2020; Scott et al., 2021). In absence of digital connectivity, students have to deal with the frustrations and consequences of not being able to connect. The adults in the participating schools recognized that, in this context, the curriculum needs to be adapted and that a key element for sustaining a collective wellbeing, is through curriculum diversification. Parents and students recalled and were thankful of the many different forms in which their school provided them with direct pedagogical support while not being able to connect online, and considered these supports as fundamental for maintaining their wellbeing. This suggests that a systems approach is needed that may address both students’ as well as teachers’, families’, and school-staffs’ collective –and not just individual- wellbeing (Alfaro et al., 2015; Harding et al., 2019; Ahmadi et al., 2020; Shirley et al., 2020). Otherwise, individual or group interventions aimed at strengthening students’ self-esteem and coping abilities, run the risk of reinforcing the “diminished” student of social and educational programs for “vulnerable” students to the detriment of collective competencies of cooperation and collaboration (Ecclestone and Rawdin, 2016).

On the other hand, a sustainable perspective of school wellbeing seeks the wellbeing of the whole school community and looks for lasting solutions over time (Hargreaves, 2002). Regarding Hargreaves and Fink. (2000) call for the breath, depth, length, diversity, and conservation of educational change, the findings from this study suggest that an ethics of multiple cares dominates within and between school actors are deeply rooted in participant’s voices and actions. Connection and caring are not just top-down human and moral values from the school principal or principal’s team but also between equal peers: between parents, between teachers, between students. Therefore, this depth is also breath in the sense that coordination is multiple and at multiple levels of the school system. However, sustainability in terms of length and conservation during and after the pandemic, are tied to issues of welfare, in this context very much linked to safeguarding an equitable access to digital connectivity which would allow students to access different forms of remote learning. The diversity of the sustainable discourse of the participants of this study, are also linked to the possibilities of offering diverse curricula and pedagogical strategies for different leaners, and in the assumption that there is no “one size fits all glove” for safeguarding a collective wellbeing. But most of all, what findings from this study suggest is that the principle of conservation of the collective/sustainable wellbeing approach during the school closure period of the COVID-19 pandemic requires policies and actions that assure digital connectivity, tightly knit coordination, and direct and indirect emotional supports that may allow teachers and school staff to not feel overwhelmed or to exhaust human energies to such levels that a collective, sustainable wellbeing is no longer possible, but on the contrary, to renew energies (Hargreaves and Fink, 2000; Hargreaves, 2002). The curricular flexibility adopted by the educational community in the four schools from this study can be understood as an expression of a sustainable approach to wellbeing, capable of questioning diversity, that is, capable of rejecting the standardization and homogenization of school processes (Hargreaves and Fink, 2000; Hargreaves, 2002). However, these school efforts should be accompanied by policies at the central, district and school levels that allow community agency, and provide means and resources for the school to articulate a collective response to the emergency according to its particular context.

### Limitations and Future Venues

This research faced various obstacles that are relevant to consider. While some correspond to an adaptation of a research design, such as the application of techniques given the context, other types of limitations are related to the scope and understanding of the phenomenon. Field work took place during the first year of the pandemic in Chile, a period that began in March 2020. This involves three key antecedents. First, the schools participating in the study were experiencing a high demand for homework because of actions to mitigate the effect of the pandemic. Secondly, the methodological design of this research tried not to increase the consequences of the pandemic; therefore, tools were selected that would facilitate the use of the times and spaces of the participants, who were not conducting in-person meetings. Thirdly, we recognize improvement in the governments’ responses concerning the research phenomenon after our phase of interviews with the participating schools, particularly with respect to the digital gap. As a consequence, a limitation that emerges is that it was not possible to carry out a participatory design that allowed collecting information and analyzing it collectively with the actors involved.

Albeit these limitations, the main contribution of this study is the evidence that, in highly unfavorable contexts of schools in contexts of poverty amidst the COVID-19, these schools can and do manage to place the collective wellbeing of the school community as a priority. This is expressed through the generation of collective spaces of aligned with an ethics of care. These spaces of collective include close coordination with students’ families. In the context of the Chilean neoliberal educational market, where parents are perceived and treated as clients of the educational service, schools usually struggle in the relationship with families (López et al., 2012). This crisis in particular has led schools to work more closely with families, to reach out to them and engage in positive relationships in order to reach the students. Findings show that this has activated many positive school-family bonding processes that protect wellbeing. Daring to innovate in different forms of relationships, they have also learned that they are capable activating support networks for the benefit of the student’s wellbeing and that of the rest of the school community, including themselves as teachers and school staff.

Future lines of research on wellbeing from a collective wellbeing and sustainability approach require new research questions concerning the modes and effects of the different forms of hybrid and in-person ways in which students have been returning to (the physical grounds of) school. This study was conducted in 2020, when vaccines were not available and the return to school was not clear. However, at the end of the 2021 school year more than 80% of the Chilean population has received two doses of vaccine including children, and schools have been operating in different forms of in-person teaching, albeit important inequities in terms of the school’s socioeconomic backgrounds: while most private schools returned at the beginning of the 2021 school year (i.e., March 2021), most public schools returned when only 4 months were left of the school year (i.e., October 2021). For the beginning of the 2022 school year starting March, the Chilean government has mandated in-person teaching with no limited classroom capacity. These different forms of “going back to school” need to form part of relevant research questions. Likewise, it is also necessary to analyze how the policies taken at the central and district levels can affect the diversity, depth and breadth of the strategies developed by the educational communities. These elements should be considered for the design, implementation, and evaluation of future educational policies and actions.

## Data Availability Statement

The datasets presented in this article are not readily available because Life Skills Program may apply some restrictions. Requests to access the datasets should be directed to VL, veronica.lopez@pucv.cl.

## Author Contributions

VL, LR, PA, JÁ, CC-A, and AMS contributed to conception and design of the study. VL and LR led the execution of the study. TC, HO, LR, and VL performed the interviews. RL-C performed and updated the literature review under the supervision of LR and VL. VL, LR, and RL-C wrote the first draft of the manuscript. PA, CC-A, JÁ, and PJ wrote the sections of the manuscript. All authors contributed to data analysis, manuscript revision, read, resubmission, and approved the submitted and resubmitted version.

## Conflict of Interest

The authors declare that the research was conducted in the absence of any commercial or financial relationships that could be construed as a potential conflict of interest.

## Publisher’s Note

All claims expressed in this article are solely those of the authors and do not necessarily represent those of their affiliated organizations, or those of the publisher, the editors and the reviewers. Any product that may be evaluated in this article, or claim that may be made by its manufacturer, is not guaranteed or endorsed by the publisher.
